# Notes and News

**Published:** 1904-01-02

**Authors:** 


### Notes and News.

The new Nightingale Ward at the Rotherham Hospital has been opened. It is the gift of the Feoffees of Rotherham and Cox who have contributed £1,150 towards the rebuilding of the hospital.

The League of Mercy has this year contributed £10,000 to King Edward's Hospital Fund for London. Its progress has been marked, and the movement is spreading in a most encouraging manner.

The ball in aid of the Grosvenor Hospital for Women and Children will be held at the Whitehall Rooms on February 15th under Royal patronage. Tickets may be had from Lady Aberdare, 83 Eaton Square, S.W.

It is to be hoped that the East London Hospital for Children will carry out the excellent proposal to establish a depôt for the supply of sterilized or modified milk for the use of infants attending the out-patient department, or in the care of local practitioners.

The British Ophthalmic Hospital at Jerusalem, is appealing for an income of £2,000. It should be unnecessary to come to England with this appeal, for Egypt is yearly visited by the wealthy of the British dominions who have special opportunity to observe the necessity for such a hospital amongst a people so afflicted by ophthalmia.

Public confidence will be increased by the evident vigilance displayed by the health authorities in inspecting the oyster supply to the Metropolis. A recent seizure has been made of oysters supplied by the well known firm Messrs. Gann, of Lower Thames Street. The oysters were condemned, and the Fishmongers' Company is pursuing investigations to discover the source of pollution, as this is not evident at the grounds whence the oysters came, which are situated on the east side of a bank called the "Spit" out of the direct scour of the River Swale.

Mr. Joseph T. Fry has made a Christmas gift of £1,000 to the Bristol General Hospital.

The Hospital for Sick Children, Great Ormond Street, has received an anonymous gift of £3,000 to endow three cots.

The late Mr. George Richmond, of Gloucester, has bequeathed £4,000 to the Samaritan Fund of St. Thomas's Hospital.

The trustees of the Annie Zunz Fund have contributed £10,000 towards the scheme to remove King's College Hospital to South London.

The new Hospital for Infectious Diseases for the Kelso district has been opened. The building has been established at a cost of about £8,000.

The city of Oporto is to have a complete system of drainage, the contract being undertaken by an English firm, Messrs. Hughes and Lancaster, of Westminster.

Two West Africans suffering from sleeping sickness have been brought to the Liverpool School of Tropical Medicine where an endeavour to cure them will be made.

The steamer *Cordoba* from Santos on arrival at Hamburgh was found to have a number of dead rats on board. On suspicion of plague the ship has been towed into quarantine pending an examination of the rats to ascertain the cause of mortality.

According to Professor Pancoast, of the University of Pennsylvania, the negro can now change his skin by the use of radium, the bleaching properties of which are now recognised. In Paris experiments with mice resulted in a new coat of white replacing the original fur after an exposure to radium.

An Emigration League has been formed under the patronage of the Duke of Sutherland, the Bishop of Ely, and others, to assist young members of large and poor families to establish themselves in Canada. The scheme appears full of good sense and promise, and it should be well supported by the patriotic. Communications should be sent to the Hon. Secretary, 4 The Causeway, East End Road, East Finchley.

We commend the attention of all hospitals to the action taken by the management of the National Hospital for the Paralysed and Epileptic in appointing a committee of ladies to report on the domestic expenditure of the institution. We are told that a saving of £300 per annum will result from the adoption of the recommendations of this committee. It might not be possible at many hospitals to reduce the domestic expenditure by anything like £300 per annum without interfering with efficiency, but in some cases it is not improbable the saving might even exceed this sum, and in any event a careful scrutiny would stimulate efforts in the right direction, and the habit of inquiry and comparison would be engendered without which all management must fall behind in efficiency.

